# Crystal structure of ruthenocenecarbo­nitrile

**DOI:** 10.1107/S205698901500540X

**Published:** 2015-03-21

**Authors:** Frank Strehler, Marcus Korb, Heinrich Lang

**Affiliations:** a Technische Universität Chemnitz, Fakultät für Naturwissenschaften, Institut für Chemie, Anorganische Chemie, D-09107 Chemnitz, Germany

**Keywords:** crystal structure, ruthenocene, ruthenocenecarbo­nitrile, sandwich compound, nitrile

## Abstract

The mol­ecular structure of ruthenocenecarbo­nitrile, [Ru(η^5^-C_5_H_4_C N)(η^5^-C_5_H_5_)], exhibits point group symmetry *m*, with the mirror plane bis­ecting the mol­ecule through the C N substituent. The Ru^II^ atom is slightly shifted from the η^5^-C_5_H_4_ centroid towards the C N substituent. In the crystal, mol­ecules are arranged in columns parallel to [100]. One-dimensional inter­molecular π–π inter­actions [3.363 (3) Å] between the C N carbon atom and one carbon of the cyclo­penta­dienyl ring of the overlaying mol­ecule are present.

## Abstract 2

The ruthenocenecarbo­nitrile mol­ecule exhibits mirror symmetry in the solid state.

## Chemical context   

The nitrile group is isoelectronic with the acetylid function (Bonniard *et al.*, 2011 ▸), which has already been investigated in electron-transfer studies (see, for example, Lang *et al.*, 2006 ▸; Poppitz *et al.*, 2014 ▸; Speck *et al.*, 2012 ▸; Hildebrandt & Lang, 2013 ▸; Miesel *et al.*, 2013 ▸). Coordination of, for example, ferrocenecarbo­nitrile towards transition metals *M* will allow investigation of the electronic properties of —C N—*M*— or —C N—*M*—N C— bridging units. A synthesis for ferrocenecarbo­nitrile has already been described in 1957 (Graham *et al.*, 1957 ▸); however, only one example of an application in electrochemical studies has been described by Dowling *et al.* (1981 ▸). This prompted us to synthesize ferrocenecarbo­nitrile transition metal complexes to investigate the electronic properties of the —C N—*M*—N C— bridging units (Strehler *et al.* 2013 ▸, 2014 ▸). In a continuation of this work, we present herein the synthesis and crystal structure of the related ruthenocenecarbo­nitrile, (I). The synthesis of this compound was realized by treatment of formyl­ruthenocene with hydroxyl­amine hydro­chloride, zinc oxide and potassium iodide in aceto­nitrile, which is similar to a procedure already described for the synthesis of ferrocenecarbo­nitrile (Kivrak & Zora, 2007 ▸).
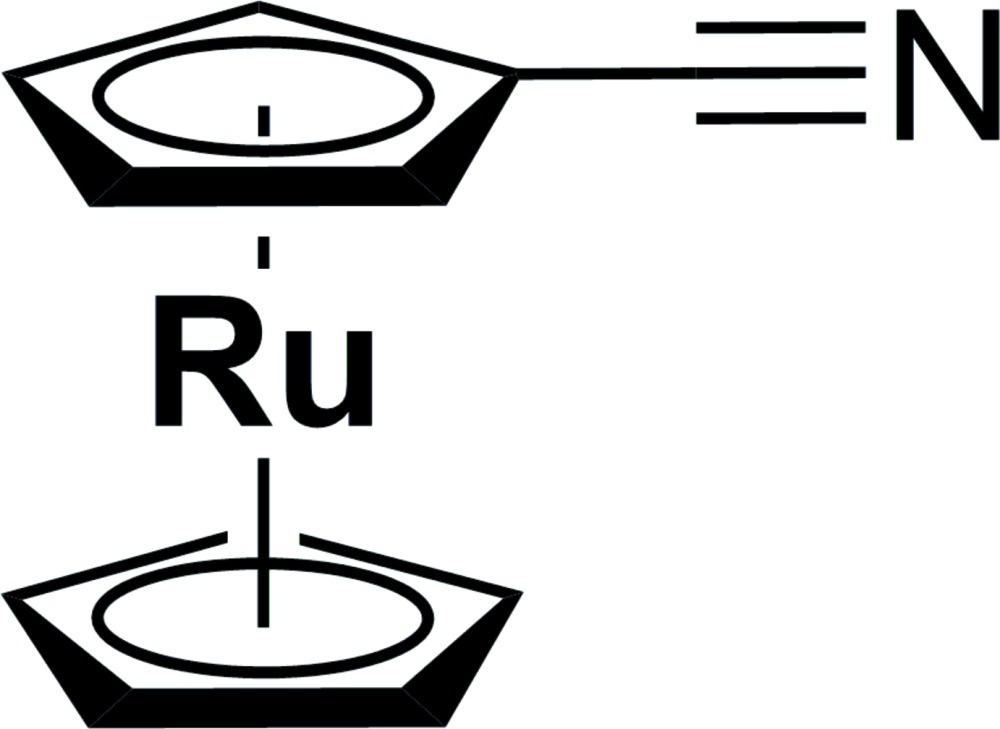


## Structural commentary   

**Figure 1:**
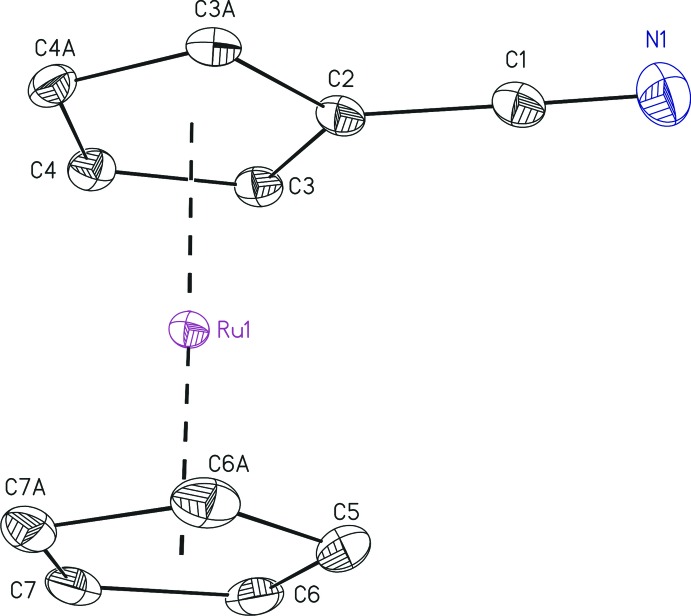
The mol­ecular structure of (I), with displacement ellipsoids drawn at the 50% probability level. All H atoms have been omitted for clarity. [Symmetry code: (A) *x*, −*y* + 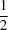, *z*.]

The title compound contains one half-mol­ecule in the asymmetric unit with a mirror plane bis­ecting the mol­ecule through atoms C1, C2, C5, N1 and Ru1 (Fig. 1 ▸). The Ru1–centroid distance to the C N-substituted cyclo­penta­dienyl ring is slightly increased [1.8179 (1) Å] compared to the unsubstituted C_5_H_5_ unit [1.8157 (1) Å]. Both cyclo­penta­dienyl rings adopt an ideally eclipsed conformation and are virtually oriented parallel towards each other, which is expressed by the bond angle at the Ru^II^ between the two centroids (= *D*), with *D*(C_5_H_4_)—Ru1—*D*(C_5_H_5_) = 178.87 (1)°. However, the Ru^II^ atom is slightly shifted from the centre of the C_5_ ring to the nitrile-bonded C2 atom, which can be explained best by the significantly different Ru—C bond lengths (Table 1 ▸) and also the Ru—*D*—C angles, which should ideally be 90° (Table 1 ▸). This is in accordance with the shift in the ferrocenedicarbo­nitrile structure (Altmannshofer *et al.*, 2008 ▸). The C N substituent itself is bent away from the metal atom in (I), with a maximum shift for N1 [0.047 (4) Å].

## Supra­molecular features   

**Figure 2:**
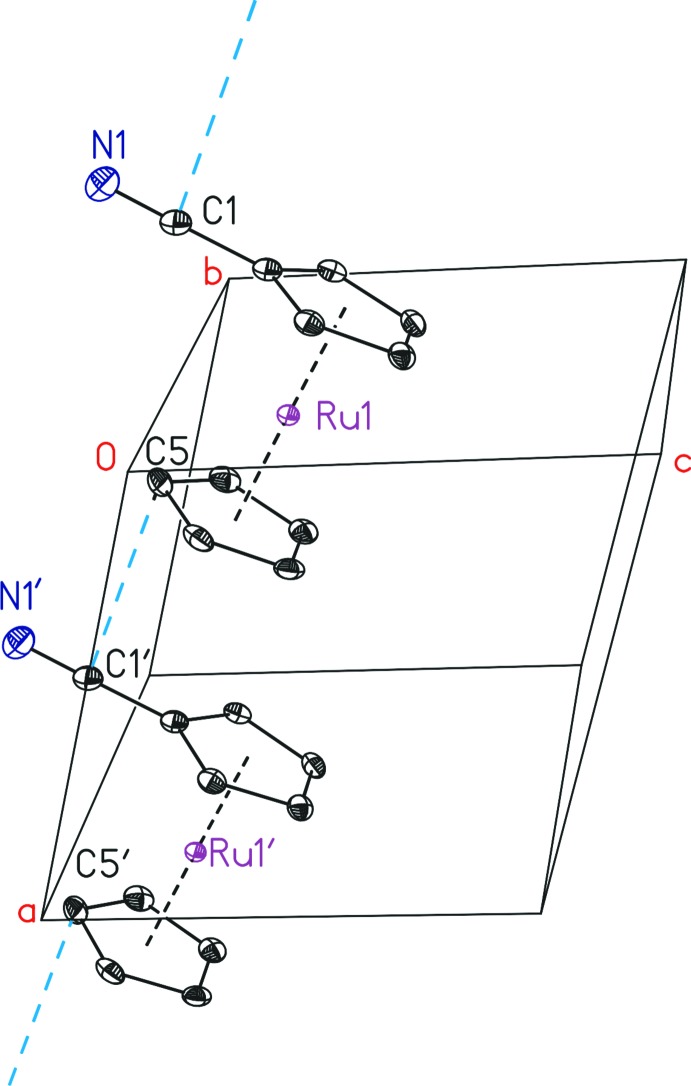
Inter­molecular π–π inter­actions (blue) between C5 and C1′ in the crystal structure of (I). All H atoms have been omitted for clarity. [Symmetry code: (′) *x* − 1, *y*, *z*.]

The packing of (I) consists of a layer-type structure parallel to (010) with the direction of the C N function aligned parallel to [10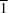], alternating between adjacent layers. A further order is observed by a columnar arrangement of slightly tilted mol­ecules parallel to [100]. Weak inter­molecular π–π inter­actions within the sum of the van der Waals radii (Σ = 3.4 Å; Bondi, 1964 ▸) are present between C5 and the C1′ atom [3.363 (3) Å] of the overlying mol­ecule in the same layer (Fig. 2 ▸).

## Database survey   

The ruthenocene backbone is hardly described in the literature. Reported derivatives contain *sp* (ethyn­yl) (Sato *et al.*, 1997 ▸; Packheiser *et al.*, 2008 ▸; Jakob *et al.*, 2008 ▸, 2009*a* ▸), *sp* ^2^ (Sato *et al.*, 1998 ▸, 2004 ▸; Jakob *et al.*, 2009*b* ▸) and *sp* ^3^ (Sokolov *et al.*, 2010 ▸; Barlow *et al.*, 2001 ▸) carbon substituents or a carb­oxy­lic acid moiety (Zhang & Coppens, 2001 ▸) and its respective Ru^II^ complex (Wyman *et al.*, 2005 ▸). They all exhibit similar Ru—*D* distances (1.795–1.823 Å) as compared to (I) [1.8179 (1)–1.8157 (1) Å] or unsubstituted ruthenocene (1.794–1.816 Å) (Ma & Coppens, 2003 ▸; Borissova *et al.*, 2008 ▸; Seiler & Dunitz, 1980 ▸).

Comparison of the C—C [1.431 (3) Å] and the C N distances [1.148 (3) Å] with the respective ferrocene carbo­nitrile derivatives (C N = 1.133–1.150; C—C = 1.428–1.433 Å; Altmannshofer *et al.*, 2008 ▸; Dayaker *et al.*, 2010 ▸; Bell *et al.*, 1996 ▸; Nemykin *et al.*, 2007 ▸; Erben *et al.*, 2007 ▸) reveals no significant influence of the central metal atom on the electronic properties of the substituent.

## Synthesis and crystallization   

Formyl­ruthenocene was prepared according to a published procedure (Mueller-Westerhoff *et al.*, 1993 ▸). Synthesis of ruthenocenecarbo­nitrile, (I): formyl­ruthenocene (2.27 g, 8.8 mmol), hydroxyl­amine hydro­chloride (0.96 g, 13.8 mmol), zinc oxide (0.86 g, 10.6 mmol) and potassium iodide (1.76 g, 10.6 mmol) were suspended in 120 ml of dry aceto­nitrile. The mixture was stirred for 4 h at precisely 368 K. After cooling the reaction mixture to ambient temperature, 18 ml of an aqueous Na_2_S_2_O_3_ (5%) solution were added in a single portion, and stirring was continued for additional 20 min. Solid particles were removed by filtration and the filtrate was extracted with ethyl acetate (3 × 50 ml). The combined organic layers were dried over MgSO_4_. All volatiles were removed under reduced pressure and the crude product was purified by flash chromatography on aluminum oxide using di­chloro­methane as eluent. Greenish crystals of (I) were obtained by slow evaporation of a saturated di­chloro­methane solution containing (I) at ambient temperature (yield: 820 mg, 3.3 mmol, 38% based on formyl­ruthenocene). IR (KBr, cm^−1^): ν = 2226 (*m*, C N), 2854 (*s*), 2925 (*s*), 3082 (*m*, C—H). ^1^H NMR (500.3 MHz, CDCl_3_, 298 K): δ 4.69 (*s*, 5H, C_5_H_5_), 4.70 (*pt*, 2H, *J*_H,H_ = 1.8 Hz), 4.70 (*pt*, 2H, *J*_H,H_ = 1.8 Hz). ^13^C{^1^H} NMR (125.7 MHz, CDCl_3_, 298 K): δ = 55.3 (C_i_-C_5_H_4_), 72.4 (C_5_H_4_), 72.9 (C_5_H_5_), 73.5 (C_5_H_4_), 119.4 (CN). HRMS (ESI–TOF, *M* ^+^): C_11_H_9_NRu: *m*/*z* = 256.9792 (calc. 256.9776).

## Refinement   

C-bonded H atoms were placed in calculated positions and constrained to ride on their parent atoms, with *U_iso_*(H) = 1.2*U*_eq_(C) and a C—H distance of 0.93 Å. Crystal data, data collection and structure refinement details are summarized in Table 2 ▸.

## Supplementary Material

Crystal structure: contains datablock(s) I. DOI: 10.1107/S205698901500540X/wm5119sup1.cif

Structure factors: contains datablock(s) I. DOI: 10.1107/S205698901500540X/wm5119Isup2.hkl

CCDC reference: 1054219

Additional supporting information:  crystallographic information; 3D view; checkCIF report

## Figures and Tables

**Table 1:** Selected bond lengths () and angles () for the clarification of the shift of the Ru1 atom towards the CN substituent in (I) *D* is the centroid of the C_5_H_4_ or C_5_H_5_ ring.

	C2	C3	C4	C5	C6	C7
Ru1C	2.1650(18)	2.1886(13)	2.2013(12)	2.1779(18)	2.1847(13)	2.1879(12)
C*D*Ru1	88.90(8)	89.63(6)	90.93(6)	89.75(9)	89.95(6)	90.16(6)

**Table 2:** Experimental details

Crystal data	
Chemical formula	[Ru(C_5_H_5_)(C_6_H_4_N)]
*M*_r_	256.26
Crystal system, space group	Monoclinic, *P*2_1_/*m*
Temperature (K)	110
*a*, *b*, *c* ()	7.2023(2), 8.6802(2), 7.2922(1)
()	106.497(2)
*V* (^3^)	437.12(2)
*Z*	2
Radiation type	Mo *K*
(mm^1^)	1.74
Crystal size (mm)	0.38 0.30 0.30

Data collection	
Diffractometer	Oxford Gemini S CCD
Absorption correction	Multi-scan (*CrysAlis RED*; Oxford Diffraction, 2006 ▸)
*T*_min_, *T*_max_	0.849, 1.000
No. of measured, independent and observed [*I* > 2(*I*)] reflections	27710, 900, 877
*R*_int_	0.019
(sin /)_max_ (^1^)	0.617

Refinement	
*R*[*F* ^2^ > 2(*F* ^2^)], *wR*(*F* ^2^), *S*	0.012, 0.032, 1.05
No. of reflections	900
No. of parameters	67
H-atom treatment	H-atom parameters constrained
_max_, _min_ (e ^3^)	0.27, 0.39

Computer programs: *CrysAlis CCD* and *CrysAlis RED* (Oxford Diffraction, 2006 ▸), *SHELXS97* and *SHELXTL* (Sheldrick, 2008 ▸), *SHELXL2013* (Sheldrick, 2015 ▸), *ORTEP-3 for Windows* and *WinGX* (Farrugia, 2012 ▸) and *publCIF* (Westrip, 2010 ▸).

## supplementary crystallographic information

### Crystal data

**Table:**

[Ru(C_5_H_5_)(C_6_H_4_N)]	*F*(000) = 252
*M**_r_* = 256.26	*D*_x_ = 1.947 Mg m^−^^3^
Monoclinic, *P*2_1_/*m*	Mo *K*α radiation, λ = 0.71073 Å
*a* = 7.2023 (2) Å	Cell parameters from 26762 reflections
*b* = 8.6802 (2) Å	θ = 3.5–28.7°
*c* = 7.2922 (1) Å	µ = 1.74 mm^−^^1^
β = 106.497 (2)°	*T* = 110 K
*V* = 437.12 (2) Å^3^	Block, yellow green
*Z* = 2	0.38 × 0.30 × 0.30 mm

### Data collection

**Table:**

Oxford Gemini S CCD diffractometer	900 independent reflections
Radiation source: fine-focus sealed tube	877 reflections with *I* > 2σ(*I*)
Graphite monochromator	*R*_int_ = 0.019
ω scans	θ_max_ = 26.0°, θ_min_ = 3.5°
Absorption correction: multi-scan (*CrysAlis RED*; Oxford Diffraction, 2006)	*h* = −8→8
*T*_min_ = 0.849, *T*_max_ = 1.000	*k* = −10→10
27710 measured reflections	*l* = −8→8

### Refinement

**Table:**

Refinement on *F*^2^	Primary atom site location: structure-invariant direct methods
Least-squares matrix: full	Secondary atom site location: difference Fourier map
*R*[*F*^2^ > 2σ(*F*^2^)] = 0.012	Hydrogen site location: inferred from neighbouring sites
*wR*(*F*^2^) = 0.032	H-atom parameters constrained
*S* = 1.05	*w* = 1/[σ^2^(*F*_o_^2^) + (0.0218*P*)^2^ + 0.1909*P*] where *P* = (*F*_o_^2^ + 2*F*_c_^2^)/3
900 reflections	(Δ/σ)_max_ < 0.001
67 parameters	Δρ_max_ = 0.27 e Å^−^^3^
0 restraints	Δρ_min_ = −0.39 e Å^−^^3^

### Special details

**Table:**

Geometry. All e.s.d.'s (except the e.s.d. in the dihedral angle between two l.s. planes) are estimated using the full covariance matrix. The cell e.s.d.'s are taken into account individually in the estimation of e.s.d.'s in distances, angles and torsion angles; correlations between e.s.d.'s in cell parameters are only used when they are defined by crystal symmetry. An approximate (isotropic) treatment of cell e.s.d.'s is used for estimating e.s.d.'s involving l.s. planes.
Refinement. Refinement of *F*^2^ against ALL reflections. The weighted *R* factor *wR* and goodness of fit *S* are based on *F*^2^, conventional *R* factors *R* are based on *F*, with *F* set to zero for negative *F*^2^. The threshold expression of *F*^2^ > σ(*F*^2^) is used only for calculating *R* factors(gt) *etc*. and is not relevant to the choice of reflections for refinement. *R* factors based on *F*^2^ are statistically about twice as large as those based on *F*, and *R* factors based on ALL data will be even larger.

### Fractional atomic coordinates and isotropic or equivalent isotropic displacement parameters (Å^2^)

**Table:**

	*x*	*y*	*z*	*U*_iso_*/*U*_eq_	
C1	−0.4152 (3)	0.2500	−0.0364 (3)	0.0162 (4)	
C2	−0.3084 (3)	0.2500	0.1617 (3)	0.0142 (4)	
C3	−0.24721 (18)	0.11497 (16)	0.27791 (19)	0.0142 (3)	
H3C	−0.2679	0.0130	0.2382	0.017*	
C4	−0.14854 (18)	0.16776 (15)	0.46603 (18)	0.0145 (3)	
H4C	−0.0935	0.1053	0.5710	0.017*	
C5	0.1429 (3)	0.2500	0.0334 (3)	0.0185 (4)	
H5C	0.0744	0.2500	−0.0957	0.022*	
C6	0.20428 (19)	0.11674 (17)	0.1491 (2)	0.0175 (3)	
H6C	0.1832	0.0149	0.1089	0.021*	
C7	0.30392 (17)	0.16746 (16)	0.33757 (19)	0.0158 (3)	
H7C	0.3591	0.1045	0.4420	0.019*	
N1	−0.5024 (2)	0.2500	−0.1949 (3)	0.0244 (4)	
Ru1	0.00474 (2)	0.2500	0.26311 (2)	0.00953 (7)	

### Atomic displacement parameters (Å^2^)

**Table:**

	*U*^11^	*U*^22^	*U*^33^	*U*^12^	*U*^13^	*U*^23^
C1	0.0120 (8)	0.0168 (9)	0.0198 (10)	0.000	0.0045 (7)	0.000
C2	0.0093 (8)	0.0168 (9)	0.0170 (9)	0.000	0.0044 (7)	0.000
C3	0.0111 (6)	0.0148 (7)	0.0182 (6)	−0.0022 (5)	0.0065 (5)	−0.0003 (5)
C4	0.0145 (6)	0.0163 (7)	0.0146 (6)	0.0003 (5)	0.0072 (5)	0.0029 (5)
C5	0.0155 (9)	0.0283 (11)	0.0145 (9)	0.000	0.0087 (7)	0.000
C6	0.0143 (6)	0.0190 (7)	0.0222 (7)	0.0005 (5)	0.0101 (5)	−0.0047 (6)
C7	0.0098 (6)	0.0191 (7)	0.0193 (6)	0.0033 (5)	0.0054 (5)	0.0029 (5)
N1	0.0222 (9)	0.0274 (10)	0.0208 (9)	0.000	0.0015 (7)	0.000
Ru1	0.00850 (10)	0.00992 (10)	0.01040 (10)	0.000	0.00305 (6)	0.000

### Geometric parameters (Å, º)

**Table:**

C1—N1	1.148 (3)	C5—Ru1	2.1780 (18)
C1—C2	1.431 (3)	C5—H5C	0.9300
C2—C3^i^	1.4401 (17)	C6—C7	1.4274 (19)
C2—C3	1.4401 (17)	C6—Ru1	2.1848 (13)
C2—Ru1	2.1649 (18)	C6—H6C	0.9300
C3—C4	1.4294 (18)	C7—C7^i^	1.433 (3)
C3—Ru1	2.1885 (13)	C7—Ru1	2.1878 (12)
C3—H3C	0.9300	C7—H7C	0.9300
C4—C4^i^	1.428 (3)	Ru1—C6^i^	2.1848 (13)
C4—Ru1	2.2013 (12)	Ru1—C7^i^	2.1878 (12)
C4—H4C	0.9300	Ru1—C3^i^	2.1885 (13)
C5—C6^i^	1.4262 (18)	Ru1—C4^i^	2.2013 (12)
C5—C6	1.4262 (18)		
			
N1—C1—C2	179.4 (2)	C5—Ru1—C6	38.16 (5)
C1—C2—C3^i^	125.52 (8)	C6^i^—Ru1—C6	63.94 (8)
C1—C2—C3	125.52 (8)	C2—Ru1—C7^i^	160.38 (4)
C3^i^—C2—C3	108.96 (16)	C5—Ru1—C7^i^	63.77 (6)
C1—C2—Ru1	123.64 (13)	C6^i^—Ru1—C7^i^	38.11 (5)
C3^i^—C2—Ru1	71.57 (8)	C6—Ru1—C7^i^	63.89 (5)
C3—C2—Ru1	71.57 (8)	C2—Ru1—C7	160.38 (4)
C4—C3—C2	106.82 (12)	C5—Ru1—C7	63.77 (6)
C4—C3—Ru1	71.48 (7)	C6^i^—Ru1—C7	63.89 (5)
C2—C3—Ru1	69.80 (9)	C6—Ru1—C7	38.11 (5)
C4—C3—H3C	126.6	C7^i^—Ru1—C7	38.23 (7)
C2—C3—H3C	126.6	C2—Ru1—C3^i^	38.63 (4)
Ru1—C3—H3C	123.8	C5—Ru1—C3^i^	127.17 (5)
C4^i^—C4—C3	108.70 (8)	C6^i^—Ru1—C3^i^	112.30 (6)
C4^i^—C4—Ru1	71.08 (3)	C6—Ru1—C3^i^	161.19 (5)
C3—C4—Ru1	70.51 (7)	C7^i^—Ru1—C3^i^	125.76 (5)
C4^i^—C4—H4C	125.7	C7—Ru1—C3^i^	159.25 (5)
C3—C4—H4C	125.7	C2—Ru1—C3	38.63 (4)
Ru1—C4—H4C	124.4	C5—Ru1—C3	127.17 (5)
C6^i^—C5—C6	108.40 (17)	C6^i^—Ru1—C3	161.18 (5)
C6^i^—C5—Ru1	71.18 (9)	C6—Ru1—C3	112.30 (6)
C6—C5—Ru1	71.18 (9)	C7^i^—Ru1—C3	159.25 (5)
C6^i^—C5—H5C	125.8	C7—Ru1—C3	125.76 (5)
C6—C5—H5C	125.8	C3^i^—Ru1—C3	64.76 (7)
Ru1—C5—H5C	123.5	C2—Ru1—C4^i^	63.69 (6)
C5—C6—C7	107.83 (13)	C5—Ru1—C4^i^	160.55 (4)
C5—C6—Ru1	70.66 (9)	C6^i^—Ru1—C4^i^	126.43 (5)
C7—C6—Ru1	71.06 (7)	C6—Ru1—C4^i^	159.58 (5)
C5—C6—H6C	126.1	C7^i^—Ru1—C4^i^	111.94 (5)
C7—C6—H6C	126.1	C7—Ru1—C4^i^	125.87 (5)
Ru1—C6—H6C	123.8	C3^i^—Ru1—C4^i^	38.01 (5)
C6—C7—C7^i^	107.97 (8)	C3—Ru1—C4^i^	63.86 (5)
C6—C7—Ru1	70.83 (7)	C2—Ru1—C4	63.69 (6)
C7^i^—C7—Ru1	70.88 (4)	C5—Ru1—C4	160.55 (4)
C6—C7—H7C	126.0	C6^i^—Ru1—C4	159.58 (5)
C7^i^—C7—H7C	126.0	C6—Ru1—C4	126.42 (5)
Ru1—C7—H7C	123.9	C7^i^—Ru1—C4	125.87 (5)
C2—Ru1—C5	113.36 (7)	C7—Ru1—C4	111.94 (5)
C2—Ru1—C6^i^	127.17 (5)	C3^i^—Ru1—C4	63.86 (5)
C5—Ru1—C6^i^	38.16 (5)	C3—Ru1—C4	38.01 (5)
C2—Ru1—C6	127.17 (5)	C4^i^—Ru1—C4	37.84 (7)
			
C1—C2—C3—C4	−179.04 (16)	C2—C3—C4—Ru1	−61.20 (10)
C3^i^—C2—C3—C4	0.13 (19)	C6^i^—C5—C6—C7	0.1 (2)
Ru1—C2—C3—C4	62.30 (9)	Ru1—C5—C6—C7	−61.69 (9)
C1—C2—C3—Ru1	118.66 (18)	C6^i^—C5—C6—Ru1	61.79 (12)
C3^i^—C2—C3—Ru1	−62.17 (12)	C5—C6—C7—C7^i^	−0.06 (12)
C2—C3—C4—C4^i^	−0.08 (12)	Ru1—C6—C7—C7^i^	−61.50 (4)
Ru1—C3—C4—C4^i^	61.12 (4)	C5—C6—C7—Ru1	61.44 (10)

Symmetry code: (i) *x*, −*y*+1/2, *z*.
